# The Btk inhibitor AB‐95‐LH34 potently inhibits atherosclerotic plaque–induced thrombus formation and platelet procoagulant activity

**DOI:** 10.1111/jth.15899

**Published:** 2022-10-17

**Authors:** Christopher W. Smith, Maan H. Harbi, Lourdes Garcia‐Quintanilla, Kieran Rookes, Helena Brown, Natalie S. Poulter, Steve P. Watson, Phillip L. R. Nicolson, Mark R. Thomas

**Affiliations:** ^1^ Institute of Cardiovascular Sciences, College of Medical and Dental Sciences University of Birmingham Birmingham UK; ^2^ Pharmacology and Toxicology Department, College of Pharmacy Umm Al‐Qura University Makkah Saudi Arabia

**Keywords:** AB‐95‐LH34, atherosclerotic plaque, glycoprotein VI, ibrutinib, platelets, thrombosis

## Abstract

**Background:**

New antithrombotic therapies with less effect on bleeding are needed for coronary artery disease. The Btk inhibitor ibrutinib blocks atherosclerotic plaque–mediated thrombus formation. However, it is associated with increased bleeding, possibly due to non–Btk‐mediated effects. Btk‐deficient patients do not have bleeding issues, suggesting selective Btk inhibition as a promising antithrombotic strategy.

**Objectives:**

To compare the antithrombotic effects of the highly selective Btk inhibitor AB‐95‐LH34 (LH34) with ibrutinib.

**Methods:**

Glycoprotein VI and G‐protein coupled receptor‐mediated platelet function and signaling were analyzed in healthy human donor platelets by lumi‐aggregometry, flow adhesion, and western blot following 1 h treatment with inhibitors in vitro.

**Results:**

LH34 showed similar inhibition of Btk‐Y223 phosphorylation as ibrutinib, but had no off‐target inhibition of Src‐Y418 phosphorylation. Similar dose‐dependent inhibition of aggregation to atherosclerotic plaque material was observed for both. However, in response to Horm collagen, which also binds integrin α2β1, LH34 exhibited less marked inhibition than ibrutinib. Both LH34 and ibrutinib inhibited platelet adhesion and aggregation to plaque material at arterial shear. Ibrutinib demonstrated the most potent effect, with complete blockade at high concentrations. Platelet activation (P‐selectin) and procoagulant activity (phosphatidylserine exposure) in thrombi were inhibited by LH34 and completely blocked by ibrutinib at high concentrations. Furthermore, plaque‐induced thrombin generation was reduced by higher concentrations of LH34 and ibrutinib.

**Conclusions:**

LH34 potently inhibits atherosclerotic plaque–induced thrombus formation and procoagulant platelet activity in vitro, with less off‐target inhibition of Src than ibrutinib, suggesting it is a promising antiplatelet therapy with the potential for reduced bleeding side effects.

**Essentials**

- Ibrutinib blocks platelet activation by plaque, but contributes to increased bleeding risk.
- Selective Btk inhibitors may block platelet activation by plaque without contributing to bleeding.
- Selective Btk inhibitor AB‐95‐LH34 inhibits glycoprotein VI–mediated platelet activation by plaque.
- Btk inhibitors reduce platelet procoagulant activity in response to plaque.

## 1 INTRODUCTION

Platelet inhibition is a crucial component of the treatment strategy for acute coronary syndromes (ACS). Dual antiplatelet therapy (DAPT) with aspirin and a P2Y_12_ antagonist reduce recurrent atherothrombotic events at the expense of increased risk of major bleeding as they target pathways common to both thrombosis and hemostasis.^1^, ^2^, ^3^ These bleeding side effects limit their use and there is also a significant risk of recurrence of atherothrombotic events in patients despite existing treatment strategies. Consequently, there remains unmet clinical need for new safe and effective long‐term therapies.^4^

New antithrombotic strategies must cause fewer hemorrhagic complications than P2Y_12_ inhibitors, while providing equivalent or superior efficacy against atherothrombosis. A promising target, platelet glycoprotein VI (GPVI), has been shown to be critical in atherothrombosis, with blockade or depletion of GPVI exhibiting protection against thrombosis in several models.^5^, ^6^, ^7^, ^8^, ^9^ Crucially, patients deficient in GPVI present with no or mild bleeding phenotype, with GPVI‐deficient mice also showing no prolongation of bleeding.^5^, ^10^, ^11^, ^12^, ^13^, ^14^ This has led to the development of GPVI inhibitors glenzocimab (humanized anti‐GPVI Fab) and revacept (dimeric GPVI‐Fc), which are currently undergoing clinical trials.^15^, ^16^ Use of these inhibitors are, however, limited by their intravenous administration, meaning they are not suitable for long‐term therapy.

Inhibition of the Tec family kinase Btk has been shown to block GPVI signaling, preferentially inhibiting thrombus formation on human atherosclerotic plaque, but not to affect integrin α2β1‐ and von Willebrand factor‐mediated adhesion to collagen, which is important for hemostasis.^17^ Repurposing oral Btk inhibitors—originally developed for the treatment of B cell malignancies—is therefore a promising strategy for long‐term inhibition of GPVI.

Bleeding side effects have, however, frequently been reported in patients treated in oncologic indications with early generation Btk inhibitors (ibrutinib, acalabrutinib, zanubrutinib, and tirabrutinb).^18^, ^19^, ^20^ While Btk inhibition clearly results in impaired platelet function, multiple studies have suggested bleeding is due to off‐target blockade of other kinases, such as Src family kinases or Tec, as no bleeding diathesis is observed in Btk‐deficient patients with X‐linked agammaglobulinemia (XLA).^21^, ^22^, ^23^, ^24^ Additional factors like disease‐specific predisposition and co‐medication also contribute to observed bleeding.

More selective Btk inhibitors that avoid these off‐target kinase effects therefore have the potential to inhibit platelet GPVI function, while avoiding increased bleeding risk. Partially enhanced selectivity for Btk was shown in kinase assays for the irreversible Btk inhibitor evobrutinib,^25^, ^26^ and thus far no bleeding events have been reported in its currently ongoing trials in patients with multiple sclerosis.^27^, ^28^ Likewise, no significant increase in severe bleeding was observed to the highly selective novel Btk inhibitor remibrutinib,^29^ currently undergoing clinical studies for chronic spontaneous urticaria ([NCT03926611]^30^ and Sjögren's syndrome [NCT04035668]), which also showed high selectivity for Btk in kinase screens.^31^

In this study, we used the covalent Btk inhibitor AB‐95‐LH34 (hereon referred to as LH34; compound 14 in Angst et al.^31^), which has a similar mechanism of action as remibrutinib, to evaluate the effect of potent and highly selective Btk inhibition on GPVI‐mediated platelet function and signaling compared to the less selective ibrutinib and evobrutinib.

## 2 METHODS

### 2.1 Materials

The α‐phospho‐tyrosine (4G10) monoclonal antibody (mAb) was from Millipore. The horseradish peroxidase (HRP)‐conjugated goat α‐rat IgG (SC 2032) and α‐Syk pAb (SC‐1077) were from Santa Cruz Biotechnology. Phospho‐specific polyclonal antibody (pAb) against pY1217 was from Cell Signaling Technology, against LAT pY200 and Btk pY223 were from Abcam, against Btk pY551 was from BD Biosciences and against Src pY418 was from Life Technologies. AB‐95‐LH34, ibrutinib (PCI‐32765), and evobrutinib were from Novartis. Eptifibatide was from GSK. The fluoroscein isothiocyanate (FITC)‐conjugated rat α‐mouse CD41 mAb was from BD Biosciences. Alexa Fluor® 647 anti‐human CD62P (P‐Selectin) antibody was from BioLegend. Annexin V Alexa Fluor 568 conjugate was from Fisher Scientific. Alexa Fluor 488‐conjugated phalloidin was from Thermo Fisher Scientific. The FITC‐conjugated rat α‐mouse, HRP‐conjugated α‐mouse, and α‐rabbit secondary pAbs and Hyperfilm enhanced chemiluminescence (ECL) autoradiography film were from Amersham Biosciences. Glucose, Bolt running buffer, Bolt antioxidant, and ECL reagent were from Thermo Fisher Scientific. Collagen related peptide (CRP) was from CambCol Ltd. Collagen (Horm, equine tendon, 95% type‐1, 5% type IV) was from Takeda. Thrombin receptor activating peptide (TRAP) was from Severn Biotech Ltd. ADP and arachidonic acid (ASPi) were from Roche Diagnostics Corporation. Non‐fatty acid free bovine serum albumin (BSA) was from First Link UK Ltd. ChronoLume® and ATP standard were from Chrono‐Log Corporation. Methanol and ethanol were from VWR Chemicals. Transblot Turbo western blotting buffer was from Bio‐Rad Laboratories. All other reagents were purchased from Sigma‐Aldrich.

### 2.2 Approvals and ethics

Ethical approval for collecting blood from healthy volunteers was granted by Birmingham University Internal Ethical Review (ERN_11–0175) in accordance with the Declaration of Helsinki. Ethical approval for use of atherosclerotic plaque material from patients undergoing carotid endarterectomy was provided by the North‐West Haydock Research Ethics Committee (20/NW/0001).

### 2.3 Blood collection

Blood was taken by venipuncture from consenting patients or healthy, drug‐free volunteers, into 4% sodium citrate (1:9, v/v).

### 2.4 Human platelet preparation

Warmed acid citrate dextrose (ACD) 1:10 (v/v) was added to citrated whole blood then centrifuged (200 × *g*, 20 min, room temperature) and platelet‐rich plasma (PRP) collected. PRP was then centrifuged (1000 × *g*, 10 min, room temperature) in the presence of 0.5 μmol/L prostacyclin (PGI_2_), and the supernatant discarded. The platelet pellet was resuspended in 24 ml modified‐Tyrode's‐HEPES buffer (134 mM NaCl, 0.34 mM Na_2_HPO_4_, 2.9 mM KCl, 12 mM NaHCO_3_, 20 mM HEPES, 5 mM glucose, 1 mM MgCl_2_; pH 7.3) and 3 ml ACD then centrifuged (1000 × *g*, 10 min, room temperature) in the presence of 0.2 μg/ml PGI_2_. Supernatant was discarded and platelet pellet resuspended in modified‐Tyrode's‐HEPES buffer to the required concentration. Platelets were rested for 30 min prior to use in any experiments

### 2.5 Light transmission aggregometry and granule secretion

Aggregation and ATP secretion in washed platelets (2 × 10^8^/ml) or PRP under stirring conditions (1200 rpm) at 37°C were measured in a lumi‐aggregometer (Model 700, ChonoLog) for 5 min. Aggregation was monitored by measuring changes in light transmission, with ChronoLume® (D‐luciferin‐luciferase mixture) added to enable simultaneous measurement of ATP release by luminescence. Inhibitors (50 nM ‐ 2 μM) or vehicle (dimethyl sulfoxide [DMSO]) were incubated with washed platelets or PRP for 1 h prior to stimulation.

### 2.6 Whole blood aggregometry

Aggregation was measured in whole blood diluted 1:1 (v:v) in warmed saline in a Multiplate multiple electrode aggregometry analyzer (Roche Diagnostics) for 5 min. Inhibitors (50 nM ‐ 2 μM) or vehicle (0.05% DMSO) were incubated with whole blood for 1 h prior to stimulation.

### 2.7 Platelet spreading

Washed platelets (2×10^7^/ml) were plated on atherosclerotic plaque coated glass coverslips for 30 min at 37°C, before non‐adherent platelets were removed and adherent platelets fixed (10% neutral buffered formalin solution, 10 min). Inhibitors (0.5 or 2 μM) or vehicle (0.02% DMSO) were incubated with washed platelets for 30 min prior to seeding. Platelets were then permeabilised, actin cytoskeleton stained with phalloidin Alexa Fluor 488, and imaged (Zeiss Axio Observer 7 epifluorescence microscope, 63X objective). A semi‐automated machine learning based analysis workflow using KNIME 3.4 (KNIME.com AG) and Ilastik 1.1.2 (University of Heidelberg) software was then used to assess area, perimeter, classification, and number of spread platelets.^32^

### 2.8 Protein phosphorylation

Washed platelets (4×10^8^/ml) were pre‐treated with 9 μM eptifibatide to block integrin αIIbβ3 activation. Agonists were added while stirring at 1200 rpm in an aggregometer at 37°C for 180 s unless stated otherwise. LH34, ibrutinib, evobrutinib (50 nM ‐ 2 μM), or vehicle (0.05% DMSO) were incubated with washed platelets for 1 h prior to stimulation. Activation was terminated with 5X sodium dodecyl sulfate (SDS) reducing sample buffer. Lysates were separated by SDS‐polyacrylamide gel electrophoresis, electro‐transferred, and western blotted. Western blots were probed with the stated antibodies and imaged using ECL autoradiography film. For analysis of levels of phosphorylation, western blot films were scanned and band intensity measured using ImageJ 1.5 (National Institutes of Health) with values normalized to those seen in vehicle‐treated platelets.

### 2.9 Atherosclerotic plaque

Atherosclerotic plaque was collected from carotid endarterectomies of 10 patients with symptomatic carotid artery stenosis (transient ischemic attack or stroke) and frozen (–80°C). Samples were then crushed into a fine powder and pooled. Phosphate buffered saline (PBS) was added to pooled powder and sonicated on ice. Plaque homogenate was then centrifuged and supernatant protein concentration determined.

### 2.10 Flow adhesion

Flow studies were performed using citrated whole blood from healthy volunteers. Hirudin‐anticoagulated whole blood from healthy volunteers was used for visualization of annexin V and P‐selectin expression. Ibidi μ‐Slide VI 0.1 slide (ibidi) were coated with 1 mg/ml atherosclerotic plaque homogenate or 200 μg/ml Horm collagen overnight at 4°C. Excess substrate was removed and the channels were blocked with 4 mg/ml BSA in PBS at 4°C. Channels were flushed with PBS immediately prior to blood perfusion. Blood was incubated with indicated Btk inhibitor or vehicle (0.05% DMSO) for 1 h prior to perfusion. DiOC_6_, Alexa Fluor 647 anti‐human CD62P (P‐Selectin) antibody or annexin V Alexa Fluor 568 conjugate were preincubated with blood for 10 min at 37 °C. Blood was then perfused over the coated channels at shear rate 1000 s^−1^ for 10 min using a PHD 2000 Syringe Pump (Harvard Apparatus). Platelet adhesion and thrombus formation were captured every 30 s with an Evos FL Auto imaging system (Life Technologies) using a 20× objective for the entire flow period. Images were processed and analyzed to measure fluorescence intensity and surface coverage area (%) using semi‐automated scripts in ImageJ software.

### 2.11 Flow cytometry

Whole blood was diluted 1:9 (v/v) in staining solution (anti‐P‐selectin and activated‐integrin αIIβ3 antibodies in PBS) then stimulated by indicated agonists 1:10 (v/v) for 20 min in the dark at room temperature. Samples were then fixed with ice‐cold 1% paraformaldehyde (PFA) and analyzed on an Accuri C6 flow cytometer (BD Biosciences). Platelets were gated using forward and side scatter. Inhibitors (20 nM ‐ 5 μM) or vehicle (DMSO) were incubated in whole blood for 1 h prior to antibody addition and stimulation.

### 2.12 Thrombin generation assay

Thrombin generation was measured as previously described.^33^ PRP was prepared by centrifuging citrated whole blood (200 × *g*, 10 min, room temperature). PRP was incubated with Btk inhibitors at 37°C for 1 h, then stimulated with recombinant tissue factor or plaque homogenate in an Immulon 2 HB 96‐well round‐bottom plate (Fisher Scientific). Measurement was carried out using the Fluoroskan Ascent plate reader (Thermo Fisher Scientific) equipped with Thrombinoscope software (Thrombinoscope, Synapse BV). Endogenous thrombin potential (ETP, nM*min) and maximum thrombin generation (peak, nM) were then measured in duplicate samples at 37°C for at least 60 min (excitation, 390 nm; emission, 460 nm).

### 2.13 Statistical analysis

All data are presented as mean ± standard error of the mean (SEM) with statistical significance taken as *p* < .05 unless otherwise stated. Graphs were plotted using GraphPad Prism 9 (GraphPad Software Inc.). Statistical analysis was performed using one or two‐way analysis of variance with corrections for multiple comparisons unless otherwise stated. All statistical analyses were performed using GraphPad Prism 9.

## 3 RESULTS

### 3.1 LH34 inhibits atherosclerotic plaque–induced GPVI signaling

**FIGURE 1:**
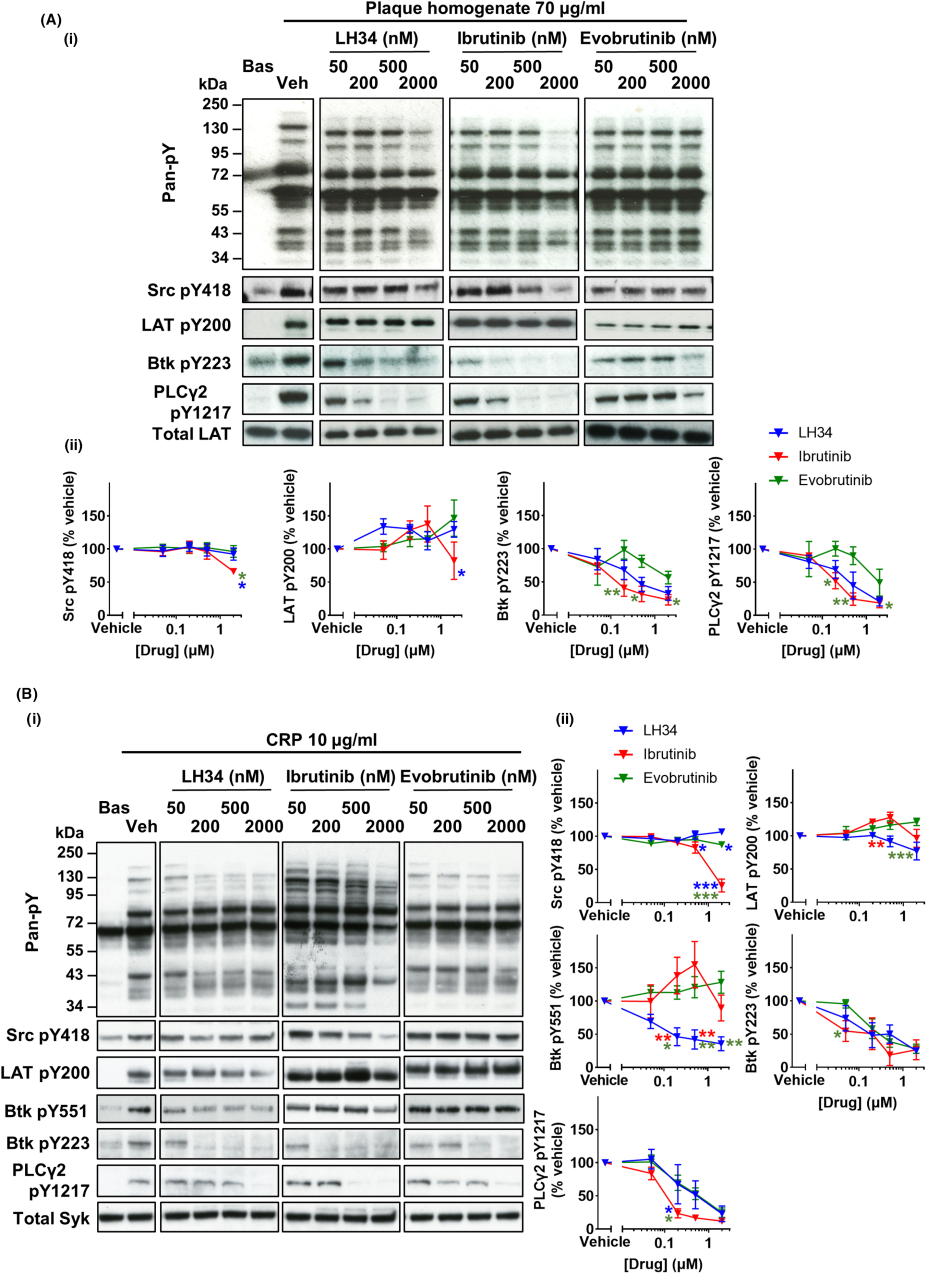
LH34 inhibits atherosclerotic plaque‐induced glycoprotein VI signaling. Healthy donor washed platelets (4 × 10^8^/ml) were incubated with vehicle (0.02% dimethyl sulfoxide [DMSO]) or indicated concentration (50, 200, 500, or 2000 nM) of Btk inhibitors LH34, ibrutinib, or evobrutinib for 1 h then stimulated with (A) plaque homogenate 70 μg/ml or (B) collagen related peptide (CRP) 10 μg/ml for 180 s in the presence of eptifibatide (9 μM) and lysed with reducing sample buffer. Whole cell lysates were then separated by sodium dodecylsulfate polyacrylamide gel electrophoresis and western blotted for tyrosine phosphorylation of indicated proteins. Total Syk and LAT were used as loading controls. (i) Representative western blots and (ii) normalized densitometry quantification. Mean ± standard error of the mean of four experiments

Previously we have shown inhibition of GPVI‐mediated aggregation by ibrutinib correlated with off‐target inhibition of Src Y418 (catalytic site) phosphorylation.^34^ We therefore assessed the effect of selective Btk inhibition on tyrosine phosphorylation downstream of GPVI following activation with atherosclerotic plaque homogenate and the synthetic GPVI‐specific agonist CRP (Figure 1).^35^ LH34 showed similar inhibition of Btk Y223 (autophosphorylation site) phosphorylation to ibrutinib, but in contrast to ibrutinib had no effect on upstream Src Y418 (Figure 1A). Evobrutinib also showed inhibition of Btk Y223 and downstream PLCγ2 Y1217 phosphorylation, but had no effect on Src Y418 phosphorylation. Similar effects were also observed to stimulation with CRP (Figure 1B), though blockade of Btk Y223 phosphorylation appears to occur at lower inhibitor concentrations. This likely reflects differences in relative strength of GPVI activation by the chosen concentrations of the different agonists. LH34 also inhibited Btk Y551 (transphosphorylation site) phosphorylation, which was not observed with the other Btk inhibitors for which phosphorylation was increased. Interestingly, increased phosphorylation of LAT Y200 was also observed with some Btk inhibitor conditions.

These reduced off‐target kinase effects of LH34 observed here compared to ibrutinib are in line with the selectivity of LH34 in kinase binding assays (Figure S1 and Table S1).

### 3.2 LH34 inhibits atherosclerotic plaque–induced platelet aggregation and dense granule release

**FIGURE 2:**
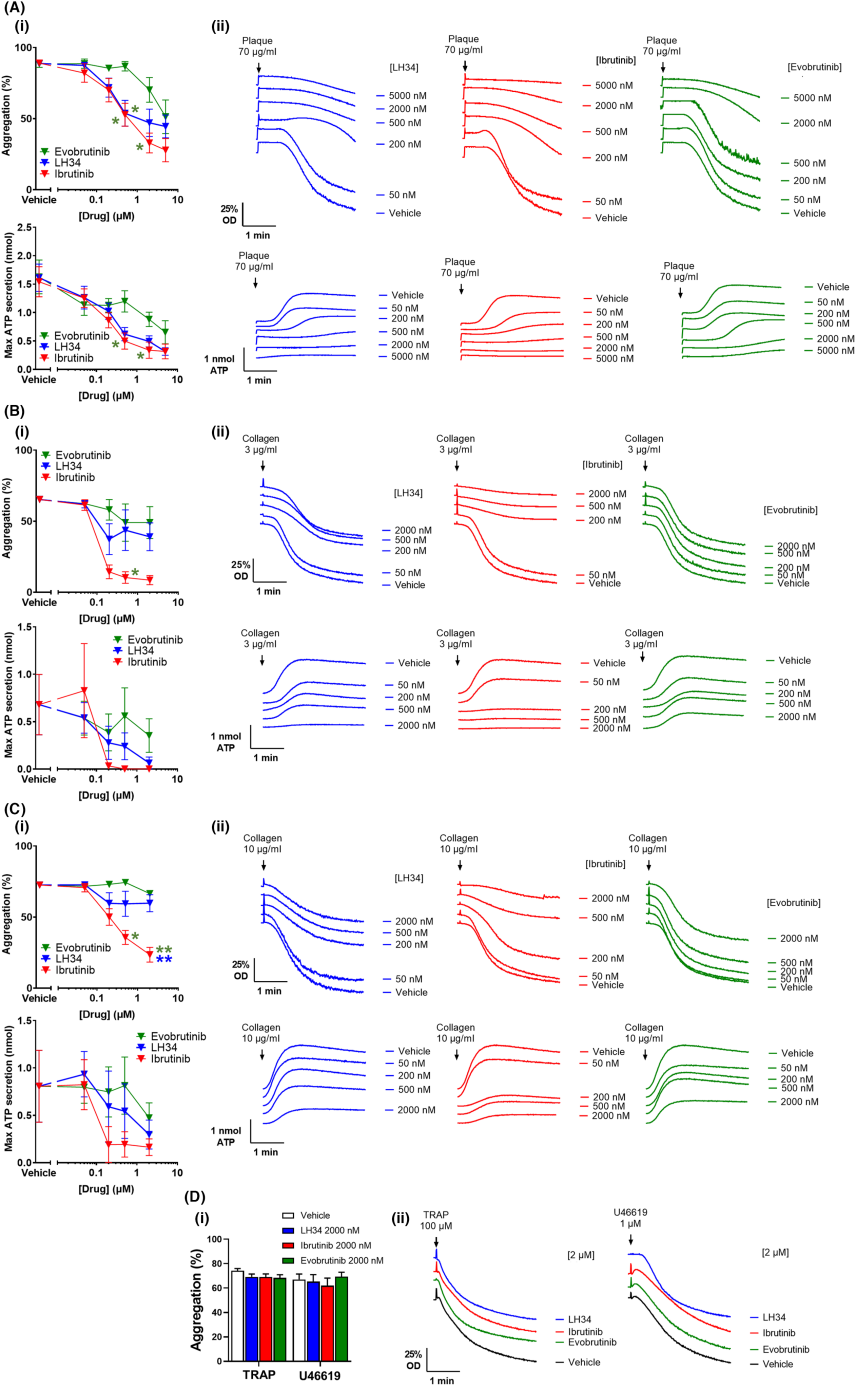
LH34 inhibits atherosclerotic plaque–induced platelet aggregation and dense granule release. Healthy donor washed platelets (2 × 10^8^/ml) were incubated with vehicle (0.02% dimethyl sulfoxide [DMSO]) or indicated concentration (50, 200, 500, or 2000 nM) of Btk inhibitors LH34, ibrutinib, or evobrutinib for 1 h before platelet aggregation and dense‐granule secretion to (A) plaque homogenate 70 μg/ml, (B) collagen 3 μg/ml or (C) 10 μg/ml, and (D) thrombin receptor activating peptide (TRAP) 100 μM or thromboxane A_2_ mimetic U46619 1 μM were measured by lumi‐aggregometry. (i) Quantification and (ii) representative traces. Mean aggregation ± standard error of the mean; *n* = 5–7. Statistical analysis by two‐way analysis of variance with Tukey's correction for multiple comparisons. *p < .05, **p < .01. Comparison indicated by color

To determine the effect of specific Btk inhibition on GPVI function, platelet aggregation and dense‐granule secretion were assessed by lumi‐aggregometry in healthy human donor washed platelets. LH34, ibrutinib, and evobrutinib showed dose‐dependent inhibition of aggregation and secretion to atherosclerotic plaque homogenate, with LH34 and ibrutinib showing greater inhibition at low concentrations than evobrutinib (Figure 2A). Isolation of the GPVI pathway, by addition of apyrase to eliminate ADP feedback, showed reduced aggregation response to plaque, with additional dose‐dependent inhibition observed to Btk inhibitors (Figure S2). In response to collagen, which also binds integrin α2β1, ibrutinib exhibited more marked inhibition than the more selective Btk inhibitors LH34 and evobrutinib (Figure 2B,C). Platelet dense‐granule secretion was more sensitive than aggregation to the effects of Btk inhibitors. No effect on G‐protein coupled receptor (GPCR)‐mediated platelet activation to TRAP or thromboxane mimetic U46619 was found to Btk inhibitors (Figure 2D). Further comparison of the rate of aggregation shows similar patterns of inhibition by Btk inhibitors as maximal aggregation (Figure S3).

**FIGURE 3:**
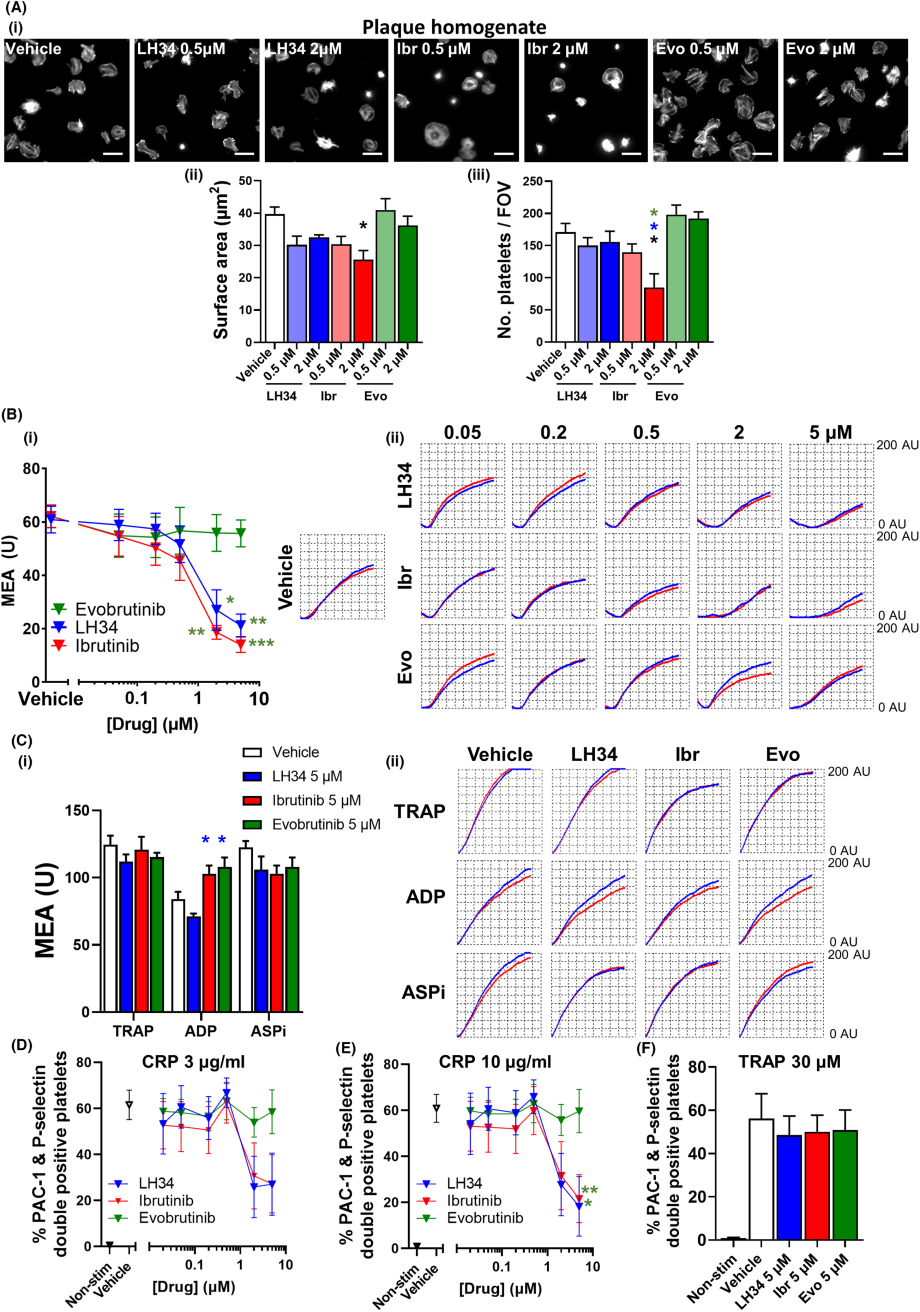
LH34 inhibits atherosclerotic plaque–induced platelet aggregation in whole blood. (A) Healthy donor washed platelets (2 × 10^7^/ml) were incubated with vehicle (0.02% dimethyl sulfoxide [DMSO]) or indicated concentration (0.5 or 2 μM) of Btk inhibitors LH34, ibrutinib, or evobrutinib for 30 min then seeded onto plaque homogenate coated coverslips and allowed to spread for 30 min at 37°C. Spread platelets were then fixed, permeabilized, and actin cytoskeleton stained. Spread platelet area was then quantified using a semi‐automated workflow as previously described.^32^ (i) Representative images, (ii) quantification of platelet area and (iii) number adherent platelets. Scale bar 10 μm. 800–1000 platelets were quantified from three different experiments. (B) Citrated healthy donor whole blood was incubated with vehicle (0.02% DMSO) or indicated concentration (0.05, 0.2, 0.5, 2, or 5 μM) of Btk inhibitors LH34, ibrutinib, or evobrutinib for 1 h. Platelet aggregation in whole blood was then measured using multiple electrode aggregometry (MEA) to plaque or (C) thrombin receptor activating peptide (TRAP; 32 μM), ADP (6.5 μM), or arachidonic acid (ASPi; 0.5 mM). (i) Quantification and (ii) representative traces. Mean ± standard error of the mean (SEM), *n* = 4–7. Platelet activation, indicated by activated integrin αIIbβ3 (PAC‐1) and P‐selectin surface expression, was assessed by flow cytometry in response to stimulation with (D) collagen related peptide (CRP) 3 μg/ml or (E) 10 μg/ml and (F) TRAP 30 μM. Mean ± SEM, *n* = 4–8. Statistical analysis in (A), (C), and (F) was by one‐way analysis of variance (anova) with Tukey's correction for multiple comparisons, all other statistical analyses by two‐way anova with Dunnett's correction for multiple comparisons. *p < .05, **p < .01, ***p < .001. Comparison indicated by color

Platelet spreading on atherosclerotic plaque was also assessed following Btk inhibition (Figure 3A). Platelets treated with low and high concentrations of LH34 exhibited a trend for reduced spreading on atherosclerotic plaque. Significant inhibition of platelet spreading was however observed to high concentration ibrutinib, which also reduced the number of adherent platelets. Evobrutinib had little effect, even at high concentrations.

### 3.3 LH34 inhibits atherosclerotic plaque–induced platelet aggregation in whole blood

Platelet aggregation to atherosclerotic plaque in whole blood was also inhibited by LH34, and showed similar dose‐dependent inhibition as ibrutinib, with evobrutinib again showing less inhibitory effect (Figure 3B). No inhibition of GPCR‐mediated aggregation was observed at high inhibitor concentrations, though ibrutinib‐ and evobrutinib‐treated platelets exhibited a slightly enhanced response to ADP compared to LH34 (Figure 3C). To further investigate this, we performed aggregations in PRP incubated for 1 h with high (2 μM) concentrations of Btk inhibitors, showing no effect on aggregation to high or low concentrations of ADP (Figure S4).

Platelet activation in response to GPVI stimulation with CRP was measured following incubation of Btk inhibitors in whole blood for 1 h. LH34 and ibrutinib inhibited platelet α‐granule secretion and integrin αIIbβ3 activation at concentrations >2 μM. Evobrutinib however had no effect (Figure 3D,E). GPCR‐mediated platelet activation to TRAP remained unaffected by Btk inhibitors (Figure 3F).

### 3.4 LH34 inhibits atherosclerotic plaque–induced platelet adhesion and thrombus development

**FIGURE 4:**
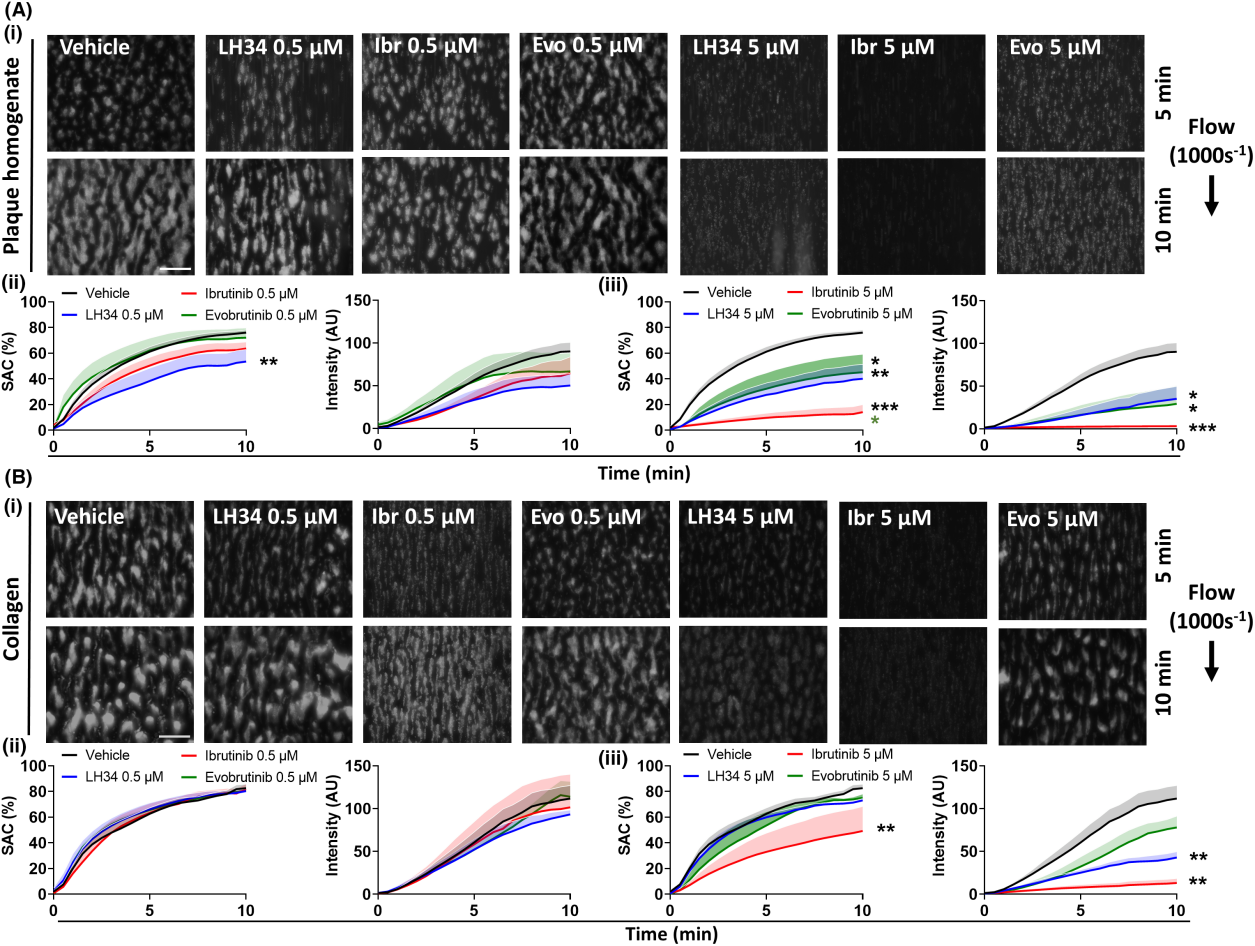
LH34 inhibits atherosclerotic plaque–induced platelet adhesion and thrombus development. Citrated healthy donor whole blood was incubated with vehicle (0.02% dimethyl sulfoxide [DMSO) or indicated concentration (0.5 or 5 μM) of Btk inhibitors LH34, ibrutinib, or evobrutinib for 1 h. Blood was then labeled with DIOC_6_ dye (10 min) and perfused over (A) human atherosclerotic plaque homogenate (1 mg/ml) or (B) Horm collagen (200 μg/ml) coated channels at 1000 s^−1^ for 10 min. (i) Representative images and (ii) quantification of platelet surface area coverage and (iii) aggregate size (fluorescence intensity) of images captured every 30 s are shown. Mean ± standard error of the mean, *n* = 3–7 per condition. Scale bar 100 μm. Statistical analyses by one‐way analysis of variance with Tukey's correction for multiple comparisons on final time point. *p < .05, **p < .01, ***p < .001. Comparison indicated by color

Ibrutinib has previously been shown to abolish platelet aggregate formation in whole blood perfused over atherosclerotic plaque homogenate, but not collagen, at arterial shear rates.^17^ Healthy donor blood was incubated with low (0.5 μM) and high (5 μM) concentrations of Btk inhibitors for 1 h before perfusion over human atherosclerotic plaque homogenate or collagen (Figure 4). Platelet adhesion (surface area coverage) and aggregate formation (fluorescence intensity) on atherosclerotic plaque homogenate was reduced by all Btk inhibitors, with ibrutinib showing more potent inhibition than LH34, demonstrating complete blockade of platelet adhesion at the high concentration (Figure 4A). No effect on platelet adhesion or aggregate size on collagen was observed at low inhibitor concentrations (Figure 4B). High concentrations (5 μM) of inhibitors however, reduced aggregate size, with ibrutinib again displaying the strongest inhibition and the only inhibitor to also reduce platelet adhesion (Figure 4B).

### 3.5 LH34 inhibits procoagulant functions of platelets that are activated by atherosclerotic plaque

**FIGURE 5:**
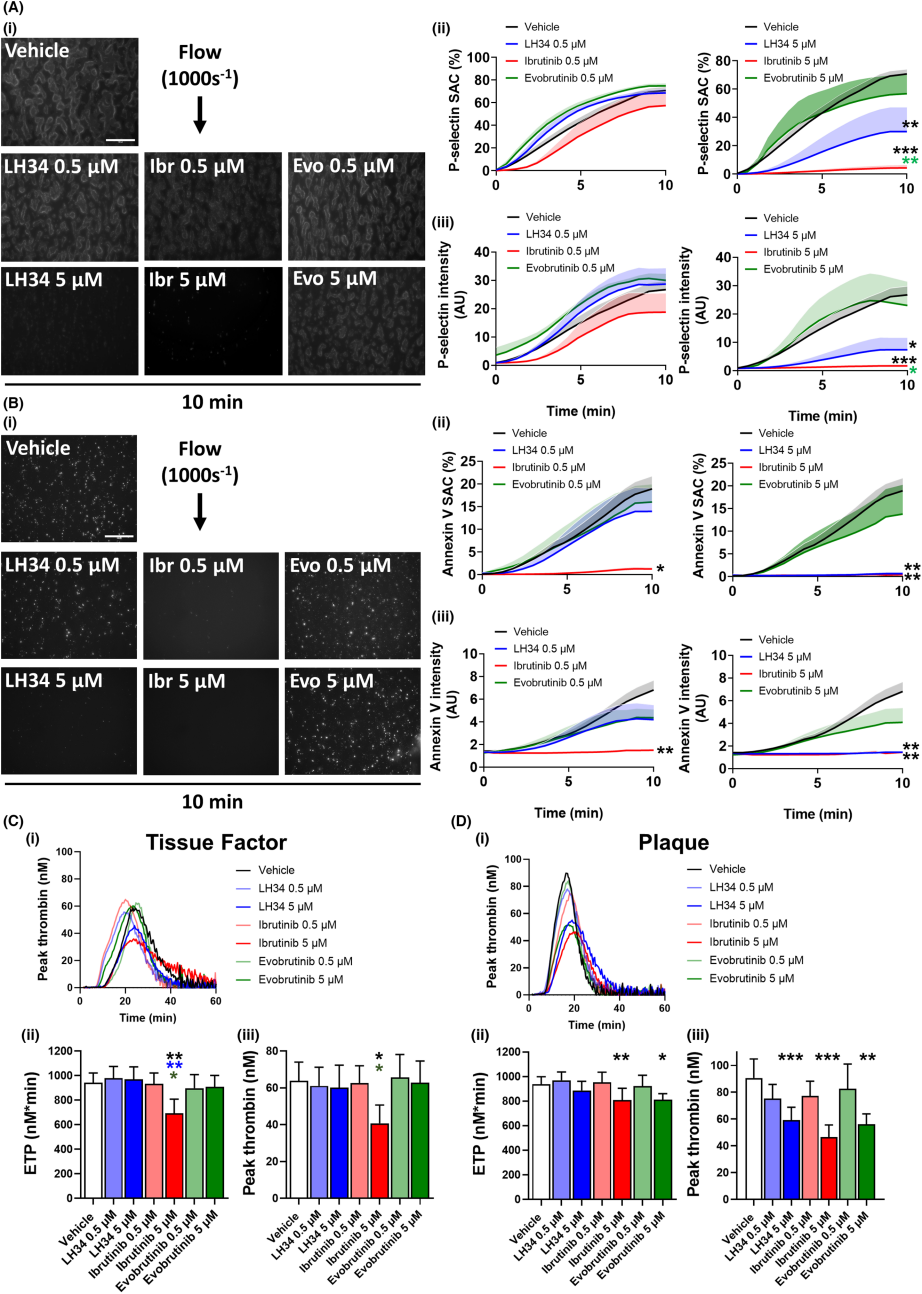
LH34 inhibits procoagulant functions of platelets that are activated by atherosclerotic plaque. Hirudin anticoagulated healthy donor whole blood was incubated with vehicle (0.02% dimethyl sulfoxide [DMSO]) or indicated concentration (0.5 or 5 μM) of Btk inhibitors LH34, ibrutinib, or evobrutinib for 1 h. Blood was then labeled with (A) P‐selectin antibody or (B) annexin V dye (10 min) and perfused over human atherosclerotic plaque homogenate (1 mg/ml) coated channels at 1000 s^−1^ for 10 min. (i) Representative images and (ii) quantification of surface area coverage and (iii) fluorescence intensity of images captured every 30 s are shown. Mean ± standard error of the mean (SEM), *n* = 3–7 per condition. Scale bar 100 μm. Statistical analyses by one‐way analysis of variance with Tukey's correction for multiple comparisons on final time point. *p < .05, **p < .01, ***p < .001. Comparison indicated by color. Thrombin generation in response to (C) tissue factor or (D) atherosclerotic plaque homogenate. (i) Representative traces and (ii) quantification of endogenous thrombin potential (ETP) and (iii) peak thrombin levels. Mean ± SEM, *n* = 7–10 per condition. Statistical analyses by mixed‐effects analysis. *p < .05, **p < .01, ***p < .001. Comparison indicated by color

In addition to looking at thrombus size, we also assessed the level of platelet activation within thrombi formed on atherosclerotic plaque (Figure 5A,B). The low concentration of Btk inhibitors (0.5 μM) showed no significant effect on distribution (surface area coverage) of P‐selectin during thrombus formation; however, high concentrations (5 μM) of LH34 resulted in significant inhibition, and complete blockade by ibrutinib (Figure 5A). Similar effects were also seen in P‐selectin intensity measurements, which were greatly reduced to high concentration LH34 and blocked by ibrutinib (Figure 5A). Evobrutinib had no effect on P‐selectin surface expression.

Procoagulant platelet formation, characterized by membrane phosphatidylserine (PS) expression, on atherosclerotic plaque was completely abolished by low and high concentrations of ibrutinib (Figure 5B). Low concentration LH34 had no effect, but high concentration LH34, similar to ibrutinib, resulted in complete blockade of procoagulant platelet formation (Figure 5B). Evobrutinib had no effect at either concentration (Figure 5B).

Btk inhibition of procoagulant activity of platelets was further assessed using a thrombin generation assay (Figure 5C). ETP and peak thrombin generation of platelets in response to tissue factor, a strong activator of procoagulant platelet function, was significantly reduced by high concentration ibrutinib, but not LH34 or evobrutinib (Figure 5C). In response to atherosclerotic plaque, ETP was reduced to high concentration of ibrutinib and evobrutinib, but not LH34 (Figure 5D). LH34 did however significantly reduce peak thrombin generation (Figure 5D).

## 4 DISCUSSION

In this study we demonstrate that highly selective Btk inhibition blocks platelet responses to atherosclerotic plaque. Platelet adhesion to collagen, which is important for physiological hemostasis, remains intact with highly selective LH34, but not ibrutinib, which may be due to off‐target inhibition of Src. We also show that Btk inhibition blocks the procoagulant functions of platelets induced by atherosclerotic plaque.

Platelet responses to atherosclerotic plaque have been shown to be mediated almost exclusively by GPVI,^9^, ^17^, ^36^, ^37^, ^38^ with anti‐GPVI antibodies, recombinant GPVI‐Fc, and GPVI‐deficient mice all shown to prevent platelet aggregate formation.^15^, ^39^, ^40^ Inhibition of platelet GPVI function by Btk inhibitors has been suggested to be due to the combined loss of Btk function and off‐target effects on Src and Tec kinases, which are also thought to be responsible for increased patient bleeding.^20^, ^22^, ^23^, ^41^ LH34 inhibited phosphorylation of Btk Y223 and downstream PLCγ2 Y1217, albeit less potently than ibrutinib, following platelet GPVI stimulation, which fits with their reported IC50 values.^31^, ^42^ LH34 however lacked the off‐target inhibition of upstream Src observed to ibrutinib. Btk Y551 phosphorylation was also inhibited by LH34, due to the inhibitor's binding mechanism. Nuclear magnetic resonance structures of the Btk full‐length apo protein show an autoinhibited conformation due to the folding of the N‐terminal regulatory domains back onto the kinase domain.^43^ While binding of ibrutinib disrupts this autoinhibited conformation, the Btk inhibitors CGI‐1746 and GDC‐0853 stabilize the autoinhibited conformation by locking the P‐loop Y551 in an inward orientation,^43^ sequestering it from activation by upstream kinases.^44^ A similar binding mode leading to an inward positioning of Y551 has been described for other inhibitors and is thought to lead to higher selectivity and cellular activity.^45^ LH34 is derived from a similar scaffold as CGI‐1746^46^ and shows a very similar binding mode resulting in Y551 sequestration.^31^ Loss of Btk Y551 phosphorylation to ibrutinib was only observed at concentrations that resulted in off‐target upstream inhibition of Src.^47^ The increased phosphorylation of LAT Y200 and Btk Y551 observed at 200–500 nM ibrutinib and 500–2000 nM evobrutinib is a curious finding, and may be due to a combination of donor variability and reagent issues, although inhibition of an inhibitory kinase such as Csk cannot be ruled out. Ibrutinib is known to inhibit Csk with a 4‐fold increased IC50 compared to Btk.^24^

Profound effects of Btk inhibition were observed to atherosclerotic plaque, with aggregation and spreading, as well as both platelet adhesion and aggregate size, reduced as previously reported.^17^, ^48^ LH34 and ibrutinib showed similar inhibition at low concentrations; however, at high concentrations, ibrutinib completely abolished platelet adhesion, and thereby aggregate formation, whereas LH34 only reduced the parameters by approximately half. This further shows off‐target effects of ibrutinib dramatically increase at higher concentrations, and suggests other components of the plaque contribute to platelet adhesion and activation.^34^, ^41^, ^49^

Less effect of Btk inhibition was observed to collagen, reflecting the involvement of integrin α2β1 in its activation of platelets. The GPVI‐specific agonist CRP however showed very similar inhibition as plaque, providing indirect evidence that Btk inhibitors block atherosclerotic plaque–mediated platelet activation by blocking GPVI. Selective Btk inhibitors LH34 and evobrutinib did not affect platelet adhesion to collagen under flow, with only high concentration ibrutinib inhibiting platelet surface area coverage. Lack of effect by more selective inhibitors shows Btk has little role in integrin α2β1 function, which is critical for platelet arrest and aggregate formation on collagen, and signifies ibrutinib has off‐target effects on this pathway.^23^, ^50^, ^51^, ^52^ Reports from other groups showed ibrutinib treatment delayed rather than reduced platelet adhesion to collagen with similar surface area coverage achieved as vehicle treatment after 5 minutes; this was however at a lower shear rate (600 s^−1^) and concentration of ibrutinib (1 μM).^17^ Thrombus formation on collagen, however, does appear to be affected by Btk inhibition, with high concentration of all inhibitors reducing aggregate size, a parameter not shown in previous studies.^17^

GPVI signaling is the major pathway for procoagulant platelet formation, which requires high sustained elevation of cytosolic Ca^2+^ from intracellular stores and extracellular influx.^53^, ^54^, ^55^, ^56^ Activation of platelet G_q_‐coupled protease‐activated receptors can also result in sustained calcium increase, but activation by secondary mediators ADP and thromboxane A_2_ alone result in weak generation of procoagulant platelets.^57^ This potentially explains why aspirin and blockade of P2Y_1_ and P2Y_12_ have only minimal effect on their formation.^57^, ^58^, ^59^ Procoagulant platelets provide the membrane requisite for prothrombinase complex assembly, resulting in localized thrombin generation and coagulation.^53^ Atherosclerotic plaque triggered platelet procoagulant activity and thrombin generation.^9^ The ability of selective Btk inhibition to block formation of adherent procoagulant platelets under flow is consistent with this being a GPVI‐mediated process. Complete blockade was not however observed in thrombin generation assays. This is likely due to the greater contribution of tissue factor and procoagulant phospholipids present in plaque material to stimulate procoagulant platelet formation in solution, whereas GPVI stimulation has been reported to be a weaker driver requiring co‐stimulation.^9^, ^53^, ^58^, ^60^, ^61^ Platelet procoagulant activity is also vital for maintaining hemostasis,^53^ so the fact thrombin generation induced by tissue factor is unaffected by selective Btk inhibition suggests that coagulation at sites of vessel injury should remain intact and therefore not exacerbate bleeding. This function of Btk inhibitors is distinct to current antiplatelet therapies, and suggests coadministration has the potential for synergistic effects.

Although not as potent as ibrutinib, LH34 still strongly inhibited plaque‐mediated platelet responses. Its high selectivity for Btk suggests it is less likely to result in bleeding, previously associated with off‐target inhibition of Src (and Tec) kinases in ibrutinib‐treated patients.^34^, ^41^, ^49^ Indeed, LH34 lacked ibrutinib's off‐target functional effects on platelet adhesion to collagen and tissue factor‐induced coagulation, both important components of hemostasis.

A recent report has however shown an increase in platelet function analyzer 200 (PFA‐200) in vitro closure time with remibrutinib.^48^ The phase I trial of remibrutinib reported mild and transient effects on hemostasis in 4 of 156 subjects with 2 subjects in the 600 mg multiple dose group with self‐limiting epistaxis not requiring intervention (both with a history of repeated epistaxis), as well as 2 subjects with hematomas in the 100 mg multiple dose group (one after an adequate trauma and one after a vessel puncture, both resolving spontaneously)^29^ and the first disclosure of the remibrutinib chronic spontaneous urticaria Phase 2b data confirmed a very favorable safety profile.^30^ We however await more robust findings over longer time periods, as the risk of bleeding may be highest upon initial treatment as was observed for ibrutinib.^62^, ^63^, ^64^, ^65^ No bleeding events have been reported in trials of fenebrutinib, a highly potent and reversible Btk inhibitor which similarly binds the inactive conformation of Btk.^66^, ^67^ Bleeding was also not reported for the reversible covalent inhibitor rilzabrutinib, which also inhibits Tec, despite being used in patients with immune thrombocytopenia who are highly susceptible to bleeding due to their low platelet counts.^68^

The present study focused on in‐vitro experimental systems relating to platelet function. While this enables dissection of mechanistic differences, the in‐vitro data has clear limitations in prediction of clinically‐relevant risk and benefit. Ex‐vivo experiments looking at the effect of ibrutinib‐treated patients on GPVI‐mediated platelet function have been performed previously^17^, ^34^ but LH34 has not been tested in patients and no evobrutinib‐treated patients were available to us. Therefore, while findings from this study indicate that highly‐selective Btk inhibitors could be developed further as antithrombotic drugs as they have minimal effect on mechanisms of hemostasis, it highlights the need of further studies in patients with coronary artery disease to determine the impact of Btk inhibition.

In conclusion, the selective Btk inhibitor LH34 shows potent inhibition of atherosclerotic plaque–induced thrombus formation without the off‐target kinase inhibition observed with ibrutinib. Additionally, Btk inhibition can limit the platelet–coagulation interplay in response to atherosclerotic plaque. This suggests highly selective Btk inhibitors could be repurposed as promising new treatments for atherothrombosis with a reduced effect on bleeding.

## AUTHOR CONTRIBUTIONS

Christopher W. Smith, Maan H. Harbi, and Phillip L.R. Nicolson designed and performed experiments, analyzed data, and wrote and revised the manuscript. Lourdes Garcia‐Quintanilla, Helena Brown, Kieran Rookes, and Natalie S. Poulter performed experiments, analyzed data, and revised the manuscript. Steve P. Watson designed experiments and revised the manuscript. Mark R. Thomas designed experiments and wrote and revised the manuscript. The manuscript has been read and approved for submission to JTH by all authors.

## CONFLICTS OF INTEREST

PLRN, MRT, and SPW have received research grants from Novartis, Principia, and Rigel Pharmaceuticals. PLRN has had honoraria from Bayer, Grifols, and Takeda. All other authors have no conflicts to report.

## FUNDING INFORMATION

This work was funded by Novartis.

## Supporting information


Table S1
Figure S1
Figure S2
Figure S3
Figure S4

## Data Availability

Study data will be made available upon reasonable request to corresponding author. Some data may not be made available due to privacy or ethical restrictions.
