# Supplementary material for: The Btk inhibitor AB‐95‐LH34 potently inhibits atherosclerotic plaque–induced thrombus formation and platelet procoagulant activity
Source: J Thromb Haemost. 2022 Oct 17;20(12):2939–52. doi: 10.1111/jth.15899 (PMC9827830; doi:10.1111/jth.15899)
Supplement: Supplementary file 1 — Table S1 Figure S1 Figure S2 Figure S3 Figure S4 [file JTH-20-2939-s001.docx]

**SUPPLEMENTAL MATERIAL**

**The Btk inhibitor AB-95-LH34 potently inhibits atherosclerotic plaque-induced thrombus formation and platelet procoagulant activity**

Christopher W. Smith^1^*, Maan H. Harbi^1,2^*, Lourdes Garcia-Quintanilla^1^, Kieran Rookes^1^, Helena Brown^1^, Natalie S. Poulter^1^, Steve P. Watson^1^, Phillip L.R. Nicolson^1^*, Mark R. Thomas^1^*

^1^Institute of Cardiovascular Sciences, College of Medical and Dental Sciences, University of Birmingham, Birmingham, B15 2TT, UK

^2^Pharmacology and Toxicology Department, College of Pharmacy, Umm Al-Qura University, Makkah, Saudi Arabia

***** Authors contributed equally to the study.

**Supplementary table 1. Binding constants (Kd in nM) of covalent BTK inhibitors for selected kinases and selectivity ratio vs off-target kinases (Kd,off‑target/Kd,BTK).**

|  |  | BTK |  | BMX | TEC | ITK | EGFR | ERBB2 | ERBB4 | JAK3 |
| --- | --- | --- | --- | --- | --- | --- | --- | --- | --- | --- |
| LH34 | K_d_ | 1.6 |  | 2200 | 650 | >10000 | >10000 | >10000 | >10000 | 9700 |
|  | Ratio | 1 |  | 1375 | 406 | >6250 | >6250 | >6250 | >6250 | 6063 |
| Ibrutinib | K_d_ | 1.9 |  | 1.6 | 1.7 | 57 | 6.9 | 1.2 | 2.4 | 37 |
|  | Ratio | 1 |  | 0.6 | 0.9 | 30 | 3.6 | 0.6 | 1.3 | 19 |
| Evobrutinib | K_d_ | 16 |  | 31 | 4.5 | 3700 | 7100 | >10000 | 5600 | >10000 |
|  | Ratio | 1 |  | 1.9 | 0.3 | 231 | 443 | >625 | 350 | >625 |


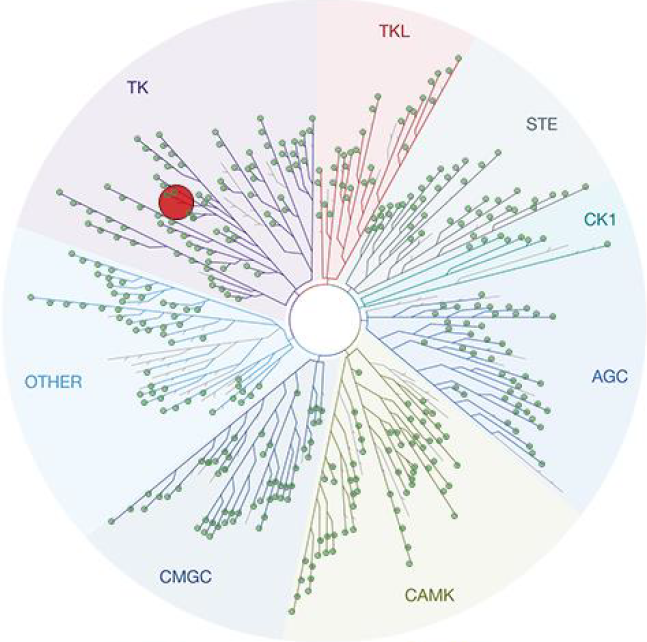


**Supplementary figure 1. TREEspot™ Kinase Dendrogram of LH34 tested at 1 µM.** Dot size indicates extent of LH34 kinase binding (percent remaining probe binding vs control). Small green dots indicate inhibition below assay-relevant 35 % threshold. BTK is marked in red and is the only kinase among the 468 tested which was inhibited by more than 35 %. Image generated using TREEspot™ Software Tool and reprinted with permission from KINOMEscan®, a division of DiscoveRx Corporation, © DISCOVERX CORPORATION 2010.


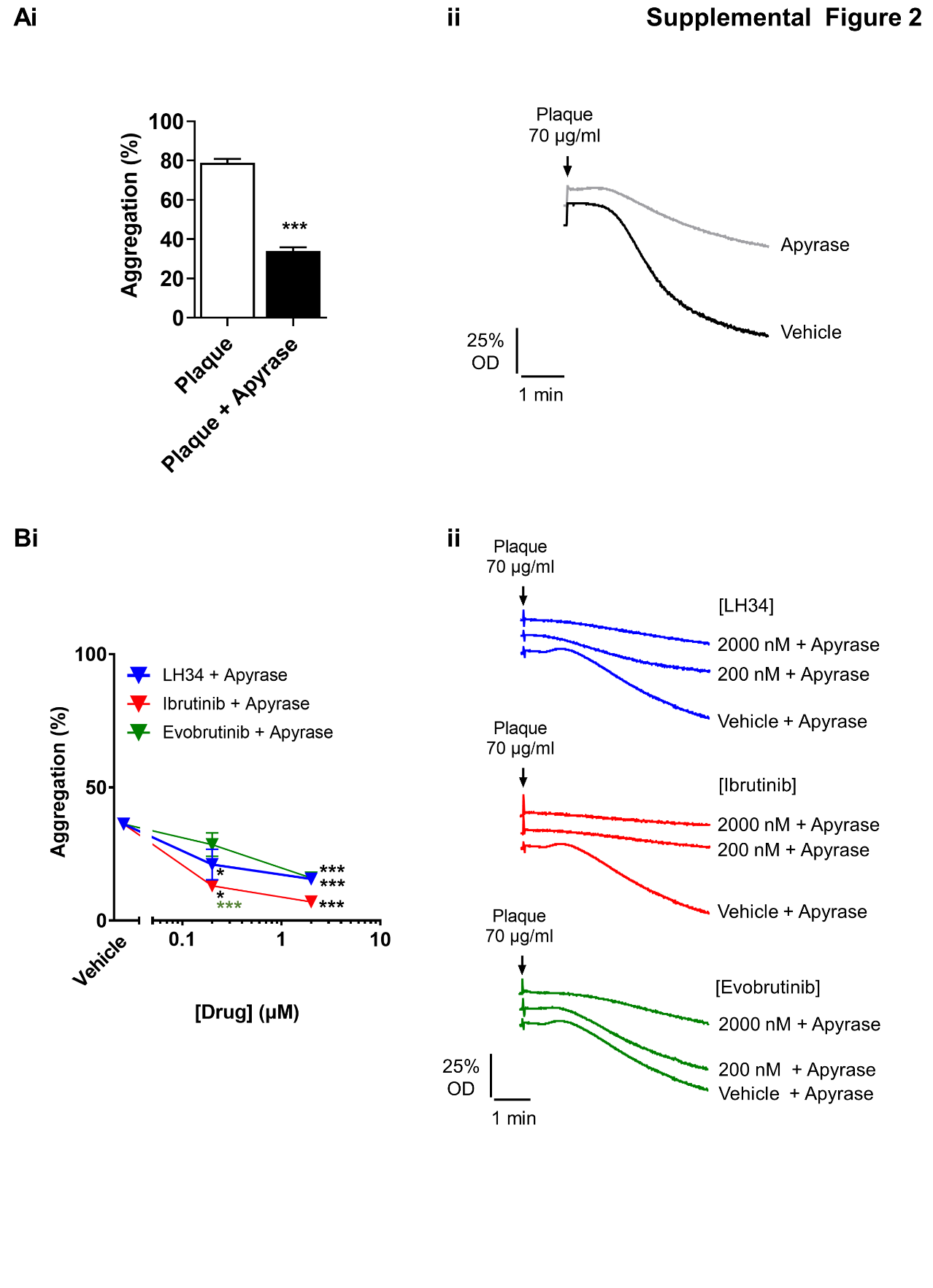


**Supplementary Figure 2. ADP feedback is important for atherosclerotic plaque-mediated aggregation.** Atherosclerotic plaque-induced aggregation was assessed in healthy donor washed platelets (2x10^8^/ml) (**A**) in the presence and absence of apyrase (1 U/ml) and (**B**) in the presence of apyrase following 1 hour incubation with Btk inhibitors LH34, ibrutinib or evobrutinib (200 or 2000 nM) or vehicle (0.02% DMSO). Plaque homogenate concentration 70 μg/ml. (**i**) Quantification and (**ii**) representative traces. Mean aggregation ± SEM; n=4-5. Statistical analysis by Student’s t test or two-way ANOVA with Tukey’s correction for multiple comparisons. *p<0.05, **p<0.0001. Comparison indicated by colour (black vs Vehicle).

**
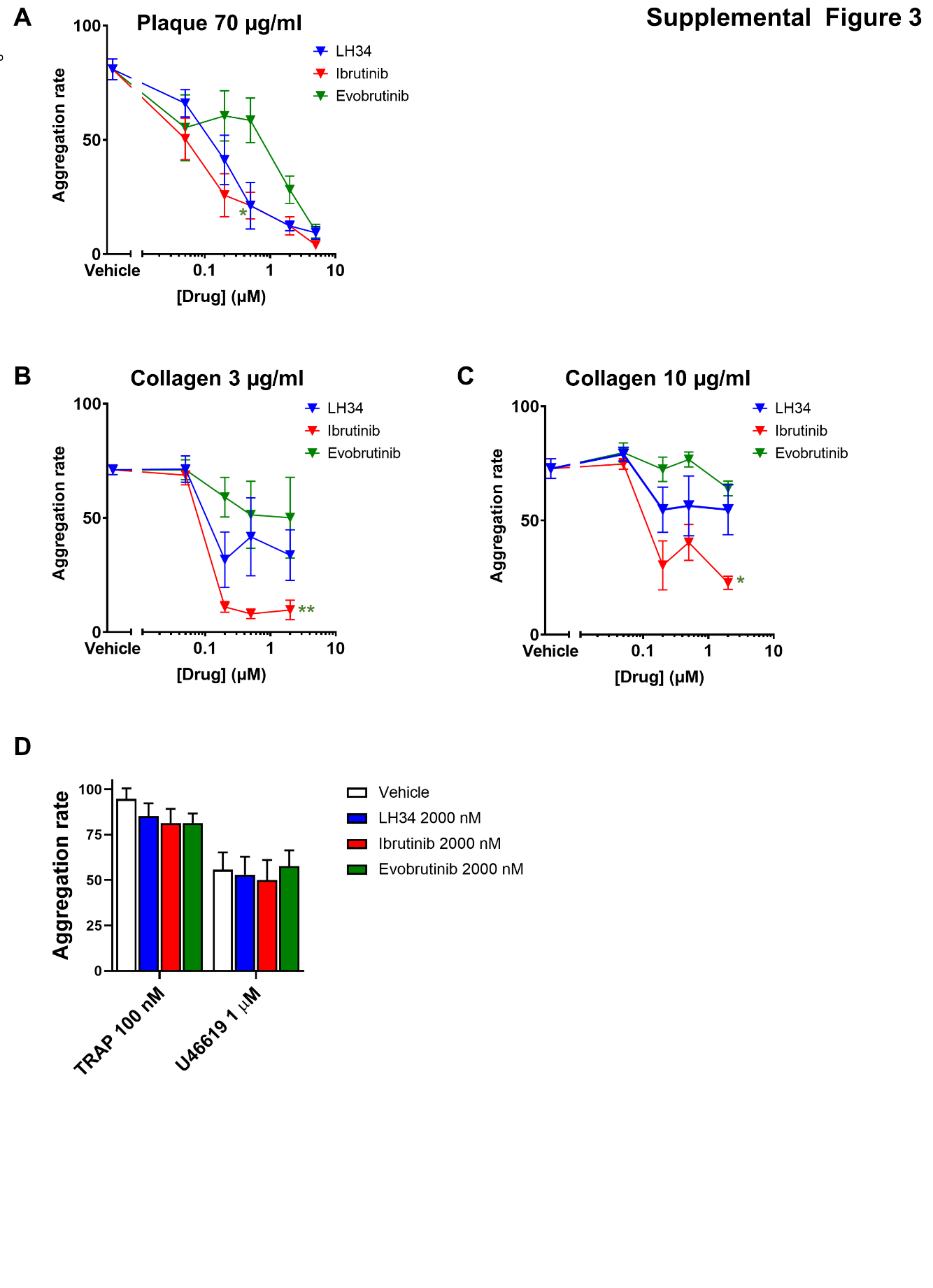
**

**Supplemental Figure 3. LH34 inhibits rate of aggregation to atherosclerotic plaque.** Healthy donor washed platelets (2x10^8^/ml) were incubated with vehicle (0.02% DMSO) or indicated concentration (50, 200, 500 or 2000 nM) of Btk inhibitors LH34, ibrutinib or evobrutinib for 1 hour before platelet aggregation to (**A**) plaque homogenate 70 μg/ml, (**B**) collagen 3 μg/ml or (**C**) 10 μg/ml and (**D**) thrombin receptor activating peptide (TRAP) 100 μM or thromboxane A_2_ mimetic U46619 1 μM were measured by lumi-aggregometry. Quantification of aggregation rate. Mean aggregation ± SEM; n=5-7. Statistical analysis by two-way ANOVA with Tukey’s correction for multiple comparisons. *p<0.05, **p<0.01. Comparison indicated by colour.


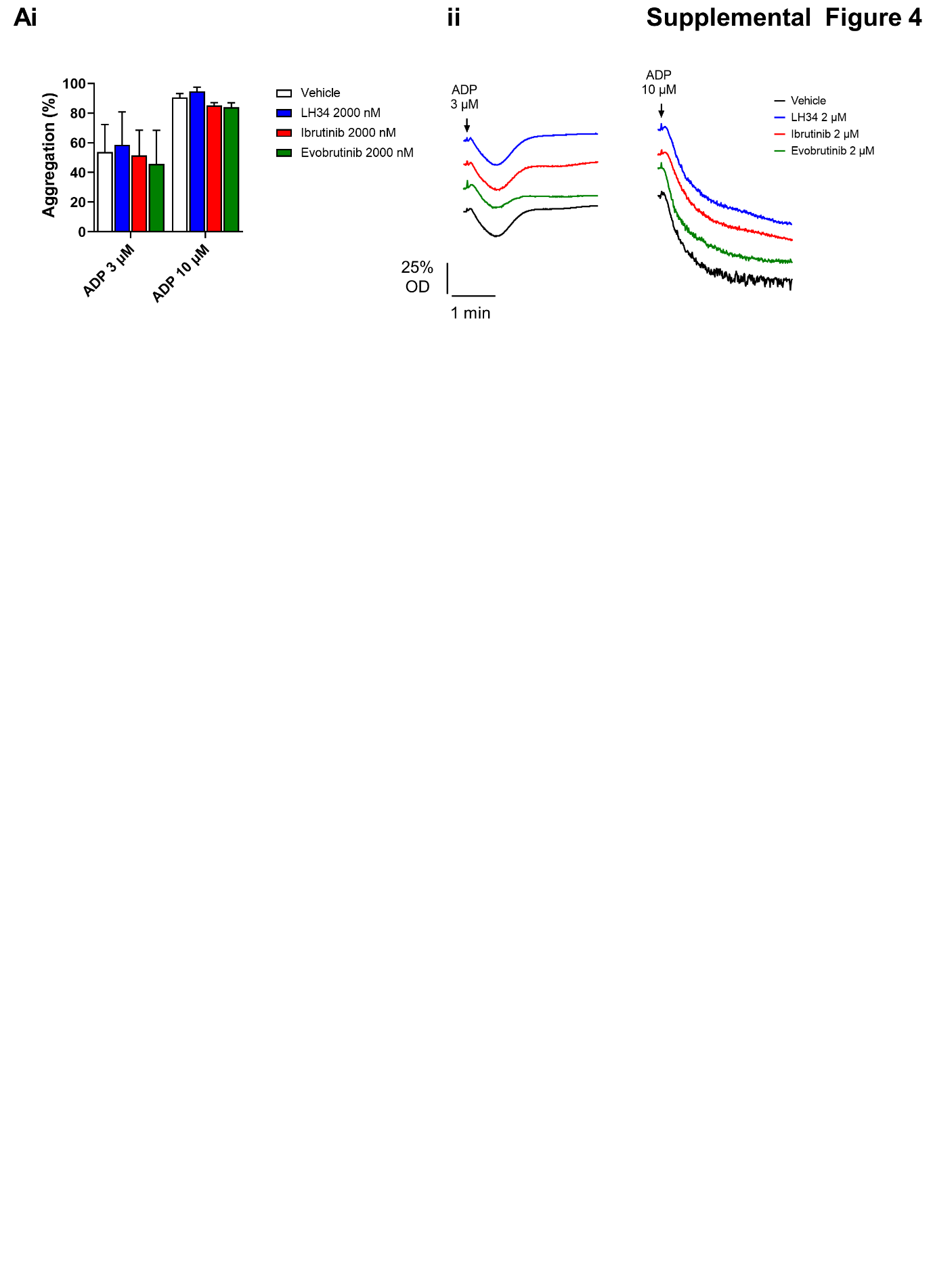


**Supplementary Figure 4. ADP-mediated platelet aggregation is unaffected by Btk inhibition in platelet rich plasma.** Aggregation induced by ADP (3 or 10 μM) was assessed by light transmission aggregometry in healthy donor platelet rich plasma incubated for 1 hour with Btk inhibitors LH34, ibrutinib or evobrutinib (2000 nM) or vehicle (0.02% DMSO). (**i**) Quantification and (**ii**) representative traces. Mean aggregation ± SEM; n=4-5. Statistical analysis by ANOVA with Tukey’s correction for multiple comparisons.
